# Characterization of a Tumor-Microenvironment-Relevant Gene Set Based on Tumor Severity in Colon Cancer and Evaluation of Its Potential for Dihydroartemisinin Targeting

**DOI:** 10.1155/2021/4812068

**Published:** 2021-06-17

**Authors:** Bo Liang, Biao Zheng, Yan Zhou, Zheng-Quan Lai, Citing Zhang, Zilong Yan, Zhangfu Li, Xuefei Li, Peng Gong, Jianhua Qu, Jikui Liu

**Affiliations:** ^1^Department of General Surgery & Carson International Cancer Research Center, Shenzhen University General Hospital/Shenzhen University Clinical Medical Academy, Shenzhen, Guangdong 518055, China; ^2^Department of Obstetrics and Gynecology & Carson International Cancer Research Center, Shenzhen University General Hospital/Shenzhen University Clinical Medical Academy, Shenzhen, Guangdong 518055, China; ^3^Department of Pharmacy, Shenzhen University General Hospital/Shenzhen University Clinical Medical Academy, Shenzhen University, Shenzhen, Guangdong, China; ^4^Department of Hepatobiliary Surgery, Peking University Shenzhen Hospital, Shenzhen, Guangdong Province, China; ^5^College of Stomatology, Dalian Medical University, Dalian, Liaoning, China

## Abstract

Colon cancer (COAD) is a leading cause of cancer mortality in the world. Most patients with COAD die as a result of cancer cell metastasis. However, the mechanisms underlying the metastatic phenotype of COAD remain unclear. Instead, particular features of the tumor microenvironment (TME) could predict adverse outcomes including metastasis in patients with COAD, and the role of TME in governing COAD progression is undeniable. Therefore, exploring the role of TME in COAD may help us better understand the molecular mechanisms behind COAD progression which may improve clinical outcomes and quality of patients. Here, we identified a Specific TME Regulatory Network including AEBP1, BGN, POST, and FAP (STMERN) that is highly involved in clinical outcomes of patients with COAD. Comprehensive *in silico* analysis of our study revealed that the STMERN is highly correlated with the severity of COAD. Meanwhile, our results reveal that the STMERN might be associated with immune infiltration in COAD. Importantly, we show that dihydroartemisinin (DHA) potentially interacts with the STMERN. We suggest that DHA might contribute to immune infiltration through regulating the STMERN in COAD. Taken together, our data provide a set of biomarkers of progression and poor prognosis in COAD. These findings could have potential prognostic and therapeutic implications in the progression of COAD.

## 1. Introduction

Colon cancer (COAD) is a common gastrointestinal cancer which is one of the leading causes of cancer deaths in the world. Although the early diagnosis and therapeutic strategy have substantially improved, the COAD-related mortality is still high [1, 2]. Current treatment options for COAD mainly contain surgery resection, radiation therapy, chemotherapy, and immunotherapy. The prognosis predictions for COAD generally rely on biomarkers with a cancer-cell-centric focus, such as the TNM staging system [3, 4]. Recent studies have pointed to the influence of the TME on the development of COAD. Thus, the TME-related factors might have the potential to serve as diagnosis and therapeutic biomarkers.

The tumor microenvironment (TME), as the niche of tumor cells, mainly contains immune infiltration cells, stromal infiltration cells, and many others [5]. Each of the components takes various roles in tumor progression. The crosstalk of tumor cells with the TME plays a crucial role in tumor progression and treatment efficacy. The impact of the TME has been extensively explored to date, including COAD. Assessment of the TME is confirmed to be a crucial biomarker for the TNM staging system of COAD [6–8]. Immune cell infiltration is proposed as a biomarker for the prognosis and contributes to clinical outcomes of COAD [4]. In the literature, the immune infiltration cells are of great prognostic value in COAD [7, 9, 10]. Furthermore, recent studies have proposed that the TME plays a crucial role in COAD development. Taken together, the TME-related factors might be a potential source of novel diagnostic, prognostic, and therapeutic biomarkers.

Dihydroartemisinin (DHA) is semiartificial synthetic derivative of artemisinin extracted from *Artemisia annua* L. [11]. In a previous study, DHA was shown to exhibit significant antitumor activity across human cancers through inhibition of cancer cell proliferation, migration, and invasion capabilities [11–15]. Although the antitumor function of DHA has been proposed recently, the precise mechanisms underlying antitumor function of DHA are still not as well understood. The role of DHA in the TME has only been reported in few studies. DHA was proposed to prevent progression of head and neck cancer via regulating macrophages in TME [16]. However, whether DHA can influence cancer progression through regulating the TME in COAD is still not well characterized. Therefore, investigation of the correlation between DHA and the TME in COAD might provide a new direction toward novel therapeutic strategies for patients with COAD.

With a goal of improving diagnosis, prognosis, and effective treatment for the patients with COAD, in the present study, we employed the bioinformatic analysis to explore the TME-related biomarkers in COAD. Our results uncovered a Specific TME Regulatory Network (STMERN) that is highly involved in clinical outcomes of patients with COAD. Furthermore, we found that DHA might contribute to progression of COAD through the STMERN. Our study proposed potential correlations among DHA, the TME, and COAD progression which might be exploited in therapeutic approaches in COAD.

## 2. Materials and Methods

### 2.1. Survival-Associated Gene Analysis

The GEPIA2 web server was used for survival analysis in COAD [17]. The most differential survival genes were calculated using survival analysis in GEPIA2. The 500 survival-associated genes were screened and arranged according to *p* values (*p* < 0.05, genes were arranged in ascending order).

### 2.2. Gene Ontology Analysis (GO)

Metascape webtool was used for GO [18]. The 500 survival-associated genes were used as input to perform GO and pathway analyses using Metascape webtool.

### 2.3. Kaplan–Meier Survival Curve

The GEPIA2 web server was used for Kaplan–Meier Survival Analysis in COAD [17]. The median value of gene expression level was used as the group cutoff.

### 2.4. Protein-Protein Interaction Analysis (PPI)

**Figure 1:**
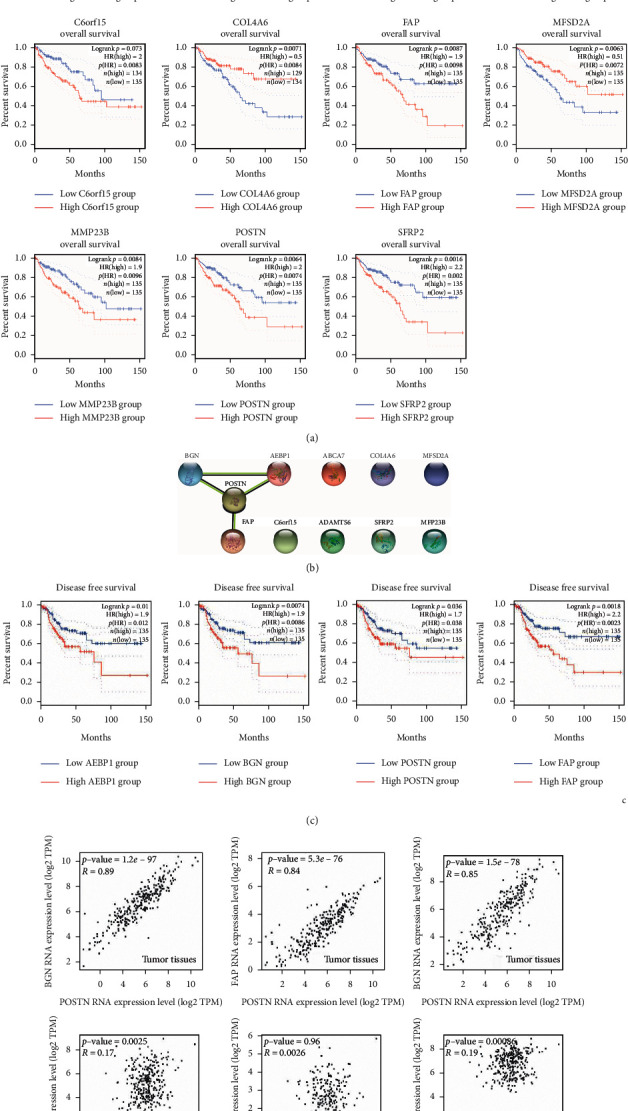
(a) Kaplan–Meier overall survival analysis of the 11 survival-associated TME genes in COAD. Differences were tested using the log-rank test. The median value of gene expression level was used as the group cutoff. Significant differences between the high-expression group and low-expression group of the 11 survival-associated TME genes in COAD were observed. (b) PPI analysis was carried out using the 11 survival-associated TME genes as the input. A PPI network was identified, including AEBP1, BGN, POSTN, and FAP. (c) Kaplan–Meier disease-free survival analysis of the STMERN in COAD. Differences were tested using the log-rank test. The median value of gene expression level was used as the group cutoff. Significant differences between the high-expression group and low-expression group of the 5 genes of the STMERN in COAD were observed. (d) Spearman' s correlation analysis among the 5 genes of the STMERN in COAD tissues and normal tissues. Significantly higher correlations were observed among the 5 genes of the STMERN in COAD tissues compared to normal tissues.

The protein-protein interactions in Figure 1(b) were predicted using multiple protein interaction function of the STRING database. The gene list contains 11 survival-associated TME genes used as input for PPI analysis.

### 2.5. Spearman's Correlation Analysis

The GEPIA2 web server was used for comprehensive gene expression analysis [17]. Spearman's correlation coefficients among POSTN, BGN, FAP, and AEBP1 expression levels in COAD were calculated using the correlation analysis function of GEPIA2.

### 2.6. Correlation between Gene Expression and Clinical Feature

**Figure 2:**
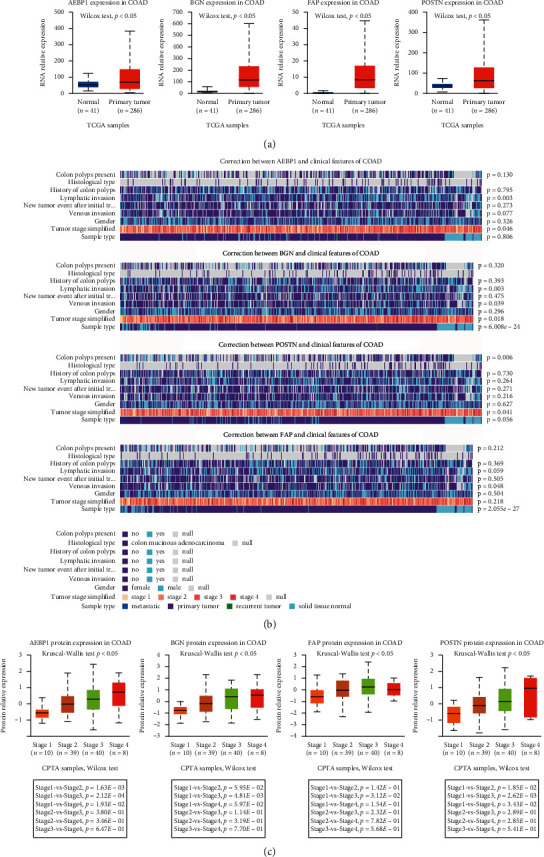
(a) RNA expression patterns of the 5 genes of the STMERN in COAD tissues and normal tissues. (b) The relationships between the RNA expression level of the STMERN and clinical features. (c) The correlations between the protein expression level of the STMERN and tumor stage.

The relationships between target genes and clinical features were examined and visualized using MEXPRESS (Figure 2(b)) and UALCAN (Figures 2(a) and 2(c)) web servers [19, 20].

### 2.7. Immune Infiltration Analysis

**Figure 3:**
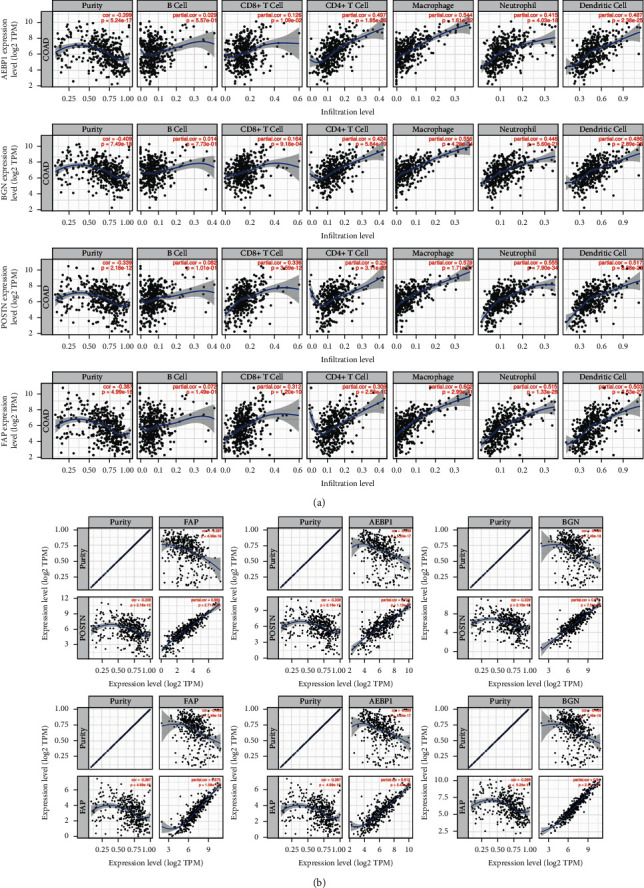
(a) Correlations between the STMERN and tumor immune infiltration cells in COAD. (b) Correlations among the genes in the STMERN.

Tumor IMmune Estimation Resource [21, 22] was used for comprehensive analysis of tumor-infiltrating immune cells. The results in Figure 3 were generated using the web server.

### 2.8. Protein-Ligand Docking

The protein-ligand docking analysis was performed using discovery studio. The protein structures of POSTN, FAP, and BGN were downloaded from the Protein Data Bank [23]. The chemical structure of dihydroartemisinin was downloaded from PubChem [24].

## 3. Results

### 3.1. Identification of a Specific TME Regulatory Network (STMERN) in COAD

**Figure 4:**
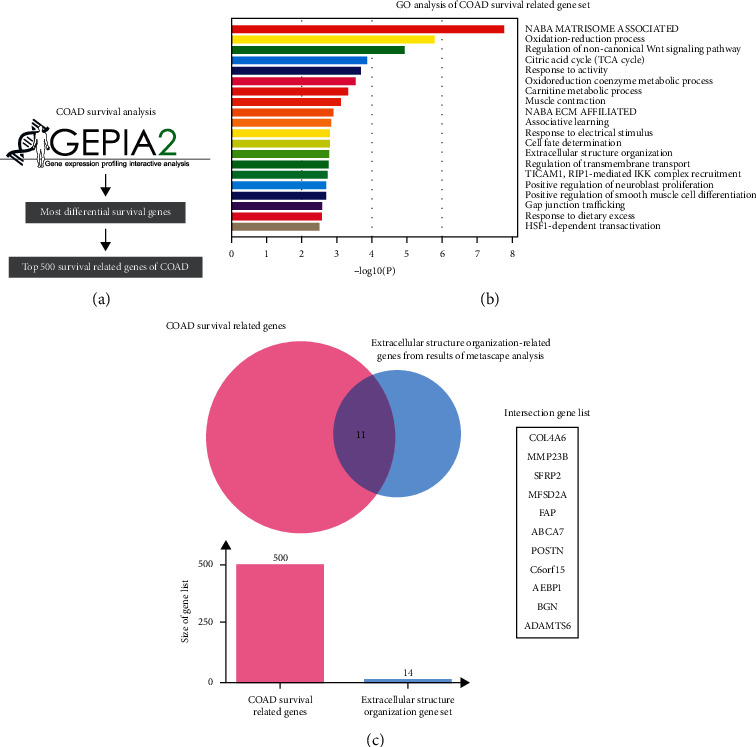
(a) The most differential survival genes were calculated in COAD using GEPIA2. The 500 survival-associated genes were screened and arranged according to *p* values. (b) GO analysis was performed using the most 500 survival-associated genes as the input. The GO terms showed that a proportion of survival-associated genes were concentrated in extracellular environment alterations which is a major structural component of the TME. (c) Venn analysis was carried out to take the intersection between survival-associated genes and extracellular structure organization-related genes from pathway hits of Metascape analysis in COAD.

To explore the relevance of biomarkers to survival in COAD, the genes with the most 500 significant association with patient survival were identified and arranged in COAD using the Most Differential Survival Genes analysis in GEPIA webtool (Figure 4(a)) [17]. To further investigate the biological roles of the survival-associated genes, we then performed gene ontology analysis. The 500 survival-associated genes were used as input to perform the GO and pathway analyses using Metascape webtool. As shown in Figure 4(b), the GO terms showed that a proportion of survival-associated genes was significantly involved in extracellular environment (NABA matrisome associated and extracellular structure organization) alterations which is a major structural component of the TME. These results revealed that the TME might contribute to survival of patients with COAD. In order to deeply explore the functions of the TME in survival of patients with COAD, we identified a set of survival-associated TME genes by taking the intersection from the COAD survival-related genes and extracellular structure organization-related genes from pathway hits of Metascape analysis. As shown in Figure 4(c), a survival-associated TME gene list containing 11 genes was identified. We then carried out Kaplan–Meier overall survival analysis to validate the correlations between 11 TME-related genes and survival of patients with COAD [17]. Significant differences in survival were observed between high-expression and low-expression groups of the 11 TME-related genes (Figure 1(a)). To further investigate the interactions among the 11 genes, we then performed Protein-Protein Interaction Assay (PPI). As shown in Figure 1(b), we identified a Specific TME Regulatory Network including AEBP1, BGN, POST, and FAP (STMERN). To further validate the correlations between the STMERN and survival of patients with COAD, the Kaplan–Meier disease-free survival was performed. We observed that higher expression of AEBP1, BGN, POST, and FAP was accompanied by worse survival of patients with COAD compared to the lower-expression group (Figure 1(c)). Next, we examined the correlations among the genes in the STMERN. The correlation coefficients were calculated among the expressions of AEBP1, BGN, POST, and FAP in COAD tissues and normal tissues. Intriguingly, as shown in Figure 1(d), we observed extremely highly positive correlations among AEBP1, BGN, POST, and FAP in COAD tissues. However, compared to COAD tissues, no significant correlations were observed in the network in normal tissues. Taken together, we identified a Specific TME Regulatory Network, which contains AEBP1, BGN, POST, and FAP (STMERN) in COAD. Meanwhile, the extremely highly positive correlations in COAD tissues compared to normal tissues suggest that the STMERN shares a common regulatory mechanism, specifically in COAD.

### 3.2. Correlations between the STMERN and Clinical Features in COAD

To further explore the clinical role of the STMERN in COAD, the expression pattern of the STMERN was examined in COAD. As shown in Figure 2(a), we examined the expression pattern of the STMERN in both COAD tissues and normal tissues using UALCAN. We observed that AEBP1, BGN, POST, and FAP were highly expressed in COAD tissues at the RNA level (Figure 2(a)) [20]. We then examined the correlations between STMERN expression and clinical features of patients with COAD using MEXPRESS [19]. We found that the STMERN RNA expression pattern was significantly correlated to COAD clinical features including lymphatic invasion, tumor stage, and tumor metastasis (Figure 2(b)). To further validate the correlations between the STMERN expression pattern and clinical features, we then examined the STMERN protein expression pattern using CPTAC analysis [20]. As shown in Figure 2(c), AEBP1, BGN, POST, and FAP expressions were highly positively correlated with tumor stage of patients with COAD at the protein level. We give a brief summary here, and we identified an overexpressed STMERN in COAD, which is highly associated with tumor severity.

### 3.3. STMERN Was Highly Involved in Immune Cell Infiltration in COAD

To further investigate the regulatory mechanism of the STMERN in TME, we examined the relationship between the STMERN and immune infiltrates using TIMER [21, 22]. We observed quite high correlations between the STMERN and immune infiltration cells (Figure 3(a)), suggesting that the STMERN was associated with immune infiltration in COAD. We then validated the correlations among the genes in the STMERN using TIMER. As shown in Figure 3(b), extremely strong correlations were observed among AEBP1, BGN, POST, and FAP expressions. We also observed a negative correlation between the STMERN and tumor purity. Our results revealed that the STMERN might contribute to the TME through regulating immune cell infiltration.

### 3.4. Dihydroartemisinin (DHA) Is Potentially Involved in COAD TME Regulating through Targeting the STMERN

**Figure 5:**
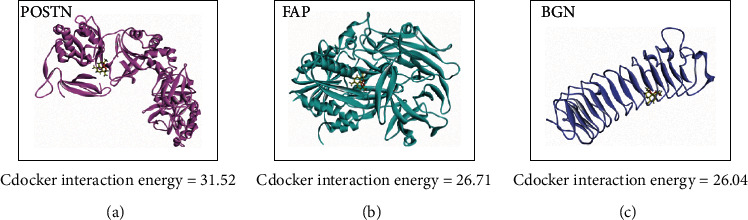
Protein ligand docking. The three-dimensional protein docking models between DHA and POSTN (a), FAP (b), and BGN (c).

DHA is a semiartificial synthetic product originated from Chinese herbal medicine. DHA has potential antitumor therapeutic effects across human cancers through inhibition of cancer cell proliferation, migration, and invasion capabilities. DHA was proposed to regulate the TME in head and neck cancer, breast cancer, and melanoma [16, 25, 26]. However, the effect of DHA in the TME of COAD has not been previously described. In the present study, we assume that DHA might contribute to COAD TME through the STMERN that we identified. To clarify and investigate the interaction between DHA and the STMERN, we performed protein-ligand docking analysis between DHA and the STMERN. The protein structures of BGN, POST, and FAP were downloaded from the PDB website. The DHA structure was downloaded from PubChem. Discovery studio was used for protein-ligand docking. As shown in Figures 5(a)–5(c), BGN, POST, and FAP showed proper docking abilities to DHA. Based on the high correlations between the STMERN and immune infiltration in our study, as well as the docking abilities between DHA and the STMERN, we suggest that DHA might regulate the TME through targeting the STMERN in COAD.

## 4. Discussion

COAD is one of the deadliest and aggressive forms of cancer in the world. The mechanism underlying progression of COAD remains unclear. Previous studies proposed that the TME could predict adverse outcomes including metastasis in patients with COAD, while the precise function of the TME in governing COAD progression is still uncertain. Thus, exploring the underlying mechanisms of the TME in COAD progression will assist us develop a more sophisticated understanding of the molecular mechanisms underlying COAD progression and, particularly, may improve clinical outcomes and survival quality of patients with COAD. In the present study, we identified a Specific TME Regulatory Network including AEBP1, BGN, POST, and FAP that is highly involved in clinical severity of COAD. Importantly, compared to normal tissues, extremely high correlations were observed among AEBP1, BGN, POST, and FAP in COAD, suggesting that the STMERN specifically formed in tumor tissues. Also, these results proposed a possibility that the formation of the STMERN in COAD might be associated with the changes of the TME in COAD compared to normal tissues. Notably, our results revealed that the STMERN was highly associated with immune infiltration in COAD. Particularly, the STMERN was highly positively correlated with tumor-associated macrophages, neutrophil, and dendritic cells. Significantly, we show that DHA potentially interacts with the STMERN. Therefore, we propose that DHA might be involved in immune infiltration via regulating the STMERN. In summary, our study provides a STMERN which contains biomarkers for severity prediction of patients. Our study provides potential prognostic and therapeutic biomarkers of progression for COAD.

Tumor-associated macrophages is a type of innate immune cells that constitute a plastic and heterogeneous cell population of the TME. Tumor-associated macrophages and their impact on the TME contribute to tumor progression and resistance to therapy [27]. Studies showed that high level of infiltration of tumor-associated macrophages is correlated with poor clinical outcomes, including prognosis and resistance to therapies [28]. Therefore, targeting tumor-associated macrophages is considered as a potential therapeutic strategy in cancer treatment. Tumor-associated dendritic cells are a type of antigen-presenting cells with crucial functions in initiating innate and adaptive immune responses [29, 30]. Recent studies have shown that tumor-associated dendritic cells contribute to cancer progression and can potentially be used as biomarkers and therapeutics [31, 32]. Tumor-associated macrophages and dendritic cells were identified as key mediators of cellular crosstalk in the TME of COAD, which can be harnessed for therapeutics development [33]. Tumor-associated neutrophils are the first response factor to inflammation and infection, which contribute to prognosis and survival, as well as correlate with progression and metastasis in cancer [34, 35]. Accumulating evidence described tumor-associated neutrophils as key drives of cancer progression via interactions with the TME [36]. Particularly, studies have shown that neutrophils are associated with prognostication and tumor severity in COAD [37–39]. It is, therefore, important to determine the mechanisms under which COAD cells initiate tumor progression through the TME. As the clinical importance of tumor-associated immune cells [4], due to the high correlations between the STMERN and tumor-associated macrophages and neutrophil, as well as dendritic cells, the STMERN might inform novel immune-centered approaches to cancer therapies.

Dihydroartemisinin (DHA) is the primary of artemisinin extracted from *Artemisia annua*, which has been extensively used in treatment of malaria. Increasing studies have indicated that DHA also exhibits anticancer activity [40, 41]. However, the precise mechanisms of DHA underlying cancer treatment are still largely unknown. Previous studies showed that DHA could inhibit COAD cell viability through regulating cell proliferation and apoptosis [15, 42].

The data presented above, in addition to many studies in the literature, indicate that DHA is involved in regulating the TME of cancer [16, 26]. However, there is no publicly available information about the role of DHA in the TME of COAD. STMERN overexpression in aggressive COAD and its correlations with immune infiltration, as well as the interactions of the STMERN to DHA, provide good prospects to our study. Not only does our work provide insights into the association of the STMERN with advanced COAD but also it holds the promise of yielding potential biomarkers and therapeutic strategies for an improved management of COAD. We proposed that DHA potentially contributes to COAD severity by changing the TME through interacting with the STMERN.

## 5. Conclusions

In the study, we identified a Specific TME Regulatory Network including AEBP1, BGN, POST, and FAP that is highly involved in clinical outcomes of patients with COAD. Furthermore, our results revealed that the STMERN might be associated with immune infiltration in COAD. Importantly, we showed that DHA potentially interacts with the STMERN. Therefore, we suggested that DHA might contribute to immune infiltration via regulating the STMERN in COAD. Our findings have therapeutic implications for the progression of COAD. These results encouraged us to further perform the studies. Future questions arising from our current study will be the direct and indirect interactions between DHA and the STMERN, and we will explore if the STMERN could be identified as a candidate pharmaceutical target in patients with COAD.

## Data Availability

The data used to support the results in this manuscript are included within the article.
